# Glass transition temperature of dried lens tissue pretreated with trehalose, maltose, or cyclic tetrasaccharide

**DOI:** 10.1186/2193-1801-3-317

**Published:** 2014-06-25

**Authors:** Tetsuhiro Kawata, Toshihiko Matsuo, Tetsuya Uchida

**Affiliations:** Department of Ophthalmology, Okayama University Medical School and Graduate School of Medicine, Dentistry, and Pharmaceutical Sciences, Okayama, 700-8558 Japan; Division of Polymer Materials Science, Faculty of Engineering and Okayama University Graduate School of Natural Science and Technology, Okayama, Japan

**Keywords:** Trehalose, Glass transition temperature, Lens, Differential scanning calorimetry, Desiccation, Maltose, Cyclic tetrasaccharide, Biological tissue, Tissue preservation, Regenerative medicine

## Abstract

**Purpose:**

Glass transition temperature is a main indicator for amorphous polymers and biological macromolecules as materials, and would be a key for understanding the role of trehalose in protecting proteins and cells against desiccation. In this study, we measured the glass transition temperature by differential scanning calorimetry of dried lens tissues as a model of a whole biological tissue to know the effect of pretreatment by trehalose and other sugars.

**Methods:**

Isolated porcine lenses were incubated with saline, 100 or 1000 mM concentration of trehalose, maltose, or cyclic tetrasaccharide dissolved in saline at room temperature for 150 minutes. The solutions were removed and all samples were dried at room temperature in a desiccator until no weight change. The dried tissues were ground into powder and placed in a measuring pan for differential scanning calorimetry.

**Results:**

The glass transition temperature of the dried lens tissues, as a mean and standard deviation, was 63.0 ± 6.4°C (n = 3) with saline pretreatment; 53.0 ± 0.8°C and 56.3 ± 2.7°C (n = 3), respectively, with 100 and 1000 mM trehalose pretreatment; 56.0 ± 1.6°C and 55.8 ± 1.1°C (n = 3), respectively, with 100 and 1000 mM maltose pretreatment; 60.0 ± 8.8°C and 59.2 ± 6.3°C (n = 3), respectively, with 100 and 1000 mM cyclic tetrasaccharide pretreatment. The glass transition temperature appeared lower, although not significantly, with trehalose and maltose pretreatments than with saline and cyclic tetrasaccharide pretreatments (*P* > 0.05, Kruskal-Wallis test). The glass transition temperature of the dried lens tissues with trehalose pretreatment appeared more noticeable on the thermogram, compared with other pretreatments.

**Conclusions:**

The glass transition temperature was measured for the first time in the dried lens tissues as an example of a whole biological tissue and might provide a basis for tissue preservation in the dried condition.

## Background

Tissue preservation in a dry condition is an ideal option for tissue storage in a long term for regenerative medicine. One key element to achieve this goal would be trehalose which is a disaccharide, formed by alpha-1,1 linkage of two D-glucose molecules. Trehalose plays a pivotal role in anhydrobiosis, biological survival under dryness, exhibited by organisms such as yeasts and seeds (Crowe et al. 1992, 1998). Trehalose has been used as food additives and medicinal additives to stabilize and hence to preserve food and drugs in a better state for a longer period of time (Richards et al. 2002). We have shown that trehalose could be used as an eyedrop to protect the corneal epithelium from dryness, caused by dry eye syndrome, including Sjogren syndrome (Matsuo 2001, 2004; Matsuo et al. 2002; Izawa et al. 2006). A current dream about the use of trehalose would be to preserve a whole tissue or cells, such as red blood cells and platelets, in the dried condition until the demand in the future (Wolkers et al. 2002).

At present, it remains basically unknown why trehalose protects cells and tissues from death under dryness. One key for explaining the effect of trehalose on the biological protection is glass transition temperature (Chen et al. 2002; Kaushik and Bhat 2003; Jain and Roy 2009). The glass transition temperature (Tg) is a temperature at which a material changes from a solid state to a glassy state. In the field of materials science, the glass transition temperature is a main indicator for polymers such as polymethylmethacrylate and acryl which are used, for example, for intraocular lenses, implanted inside the eyeball at cataract surgery. Thermal analysis was applied to the characterization of isolated proteins to measure the glass transition temperature as well as the melting temperature (Chen and Oakley 1995; Bell and Hageman 1996; Lee and Wand 2001; Hinz and Schwarz 2001; Brownsey et al. 2003; Ciesla and Vansant 2010). The biological tissues consist of many kinds of macromolecules, in other words, biopolymers such as proteins, lipids, and glycosaminoglycans. In theory, each biopolymer has its own glass transition temperature, and thus, glass transition temperature of a biological tissue is assumed to be measured to some extent as the collection of these biopolymers.

The stabilizing effect of trehalose on isolated proteins has been studied by thermal analysis, using differential scanning calorimetry (Mazzobre and Buera 1999; Attanasio et al. 2007; Hedoux et al. 2009). So far, however, no preceding study exists to measure the glass transition temperature of a biological tissue as a whole. In this study, we tried to measure the glass transition temperature of dried lens tissues by differential scanning calorimetry (Giancola 2008) in the presence or the absence of trehalose, maltose, or cyclic tetrasaccharide (Matsuo 2005) to know the effects of these sugars on the glass transition temperature.

## Results

**Figure 1:**
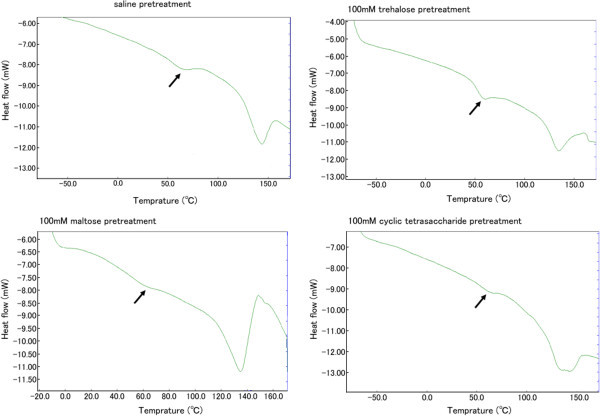
**Differential scanning calorimetric curve (thermogram) of dried porcine lens tissues, pretreated with saline, 100 mM trehalose, 100 mM maltose, or 100 mM cyclic tetrasaccharide for 150 minutes.** An arrow on each curve indicates a point of glass transition. Note that the point of glass transition, namely, glass transition temperature, appears lower, although not significantly, and more noticeable on the thermogram, obtained with trehalose pretreatment.

Thermograms (Figure 1) are shown for dried lens tissues with saline pretreatment, and pretreatment with 100 mM trehalose, 100 mM maltose, or 100 mM cyclic tetrasaccharide. The glass transition temperature of the dried lens tissues with saline pretreatment was 63.0 ± 6.4°C (n = 3) as a mean and standard deviation; the glass transition temperature of the dried lens tissues after pretreatment with 100 mM and 1000 mM trehalose was 53.0 ± 0.8°C and 56.3 ± 2.7°C (n = 3), respectively; the glass transition temperature of the dried lens tissues after pretreatment with 100 mM and 1000 mM maltose was 56.0 ± 1.6°C and 55.8 ± 1.1°C (n = 3), respectively; the glass transition temperature of the dried lens tissues after pretreatment with 100 mM and 1000 mM cyclic tetrasaccharide was 60.0 ± 8.8°C and 59.2 ± 6.3°C (n = 3), respectively. The glass transition temperature appeared lower, although not significantly, with trehalose and maltose pretreatments than with saline and cyclic tetrasaccharide pretreatments (*P* > 0.05, Kruskal-Wallis test). The glass transition temperature of the dried lens tissues with trehalose pretreatment appeared more noticeable on the thermogram, compared with other pretreatments (Figure 1).

## Discussion

The goal of this study is to measure the glass transition temperature of a biological tissue as a whole and to analyze the effect of trehalose and other sugars on the measurement of the glass transition temperature. The effect of trehalose on isolated proteins was studied by differential scanning calorimetry from the viewpoint of glass transition temperature (Mazzobre and Buera 1999; Attanasio et al. 2007; Hedoux et al. 2009). Those studies demonstrated that trehalose raised the melting temperature of lyophilized proteins but showed ambiguous results regarding the glass transition temperature (Mazzobre and Buera 1999; Attanasio et al. 2007; Hedoux et al. 2009). To the best of our knowledge, there has been no preceding report to measure the glass transition temperature of a biological tissue as a whole. We chose porcine lens as a model biological tissue to measure the glass transition temperature since the lens is an isolated small tissue in the eyeball and can be easily removed from the other surrounding tissues.

As a first step to understand the mechanism underlying the protection of a biological tissue by trehalose, we measured the melting temperature of the dried lens tissue under the presence or the absence of trehalose or cyclic tetrasaccharide in our previous study (Sun et al. 2011). That study was unique in that the melting temperature of a biological tissue as a whole, even in the dried condition, was measured by differential scanning calorimetry (Sun et al. 2011). In that study, the presence of trehalose, but not cyclic tetrasaccharide, led to an elevation of the melting temperature of the dried lens tissue (Sun et al. 2011). As an ideal condition of experiments, the melting temperature should be measured in the natural state of a biological tissue with water. However, the presence of water leads to the evaporation of water in the process of rising temperature, and hence, the evaporation hinders the measurement of the melting temperature in an accurate manner.

In the present study, we tried to measure the glass transition temperature of a biological tissue as a whole. The glass transition temperature is more difficult to measure than the melting temperature because of the subtlety of the glass transition. To overcome this limitation of the subtlety in measuring the glass transition temperature, the amount of the dried lens tissue, placed in a platform pan for the measurement, was increased about twice, compared with the amount used for the measurement of the melting temperature in the previous study (Sun et al. 2011). We could successfully measure the glass transition temperature of the dried lens tissue under this condition for the first time.

We chose maltose as a control ingredient for the pretreatment before drying of the lens tissue since maltose is another disaccharide with different bonding sites between two glucose molecules, compared with trehalose. We also chose cyclic tetrasaccharide as the pretreatment because we used this substance in the measurement of melting temperature of the dried lens tissue in the previous study (Sun et al. 2011). The pretreatment with trehalose did not lead to significant changes in the glass transition temperature, compared with saline pretreatment or pretreatment with maltose or cyclic tetrasaccharide. No significant change in the glass transition temperature of the dried lens tissue with trehalose pretreatment in the present study is in marked contrast with the elevation in the melting temperature by trehalose pretreatment in the previous study (Sun et al. 2011).

The concentration of trehalose and other sugars was set at 100 mM, based on the preceding studies to show the protective effect on cells in culture (Matsuo 2001) and also on the isolated lens tissues (Matsuo 2005). We also tested 10-times higher concentration of trehalose and other sugars, set at 1000 mM, as the pretreatment to expect better detection of the glass transition temperature. Trehalose at 1000 mM concentration was also used in thermal analysis of isolated proteins (Attanasio et al. 2007). The present study showed no difference between the different concentrations of the pretreatment with the sugars. As far as the results in this study were concerned, we could not prove the hypothesis that the stabilizing effect of trehalose on a biological tissue under the dried condition be underlain by changes in the glass transition temperature of the tissue.

The reason why the pretreatment with trehalose did not change the glass transition temperature of the dried lens tissue remains unknown at present. Based on our previous study to determine the melting temperature, the ratio of glass transition temperature over melting temperature (Tg/Tm) of the dried lens tissue falls in the range between 0.5 and 0.75 of the ratio of Tg/Tm of most polymers, as a rule of thumb in materials science. In our previous study, the melting temperature of the dried lens tissue was elevated by about 10°C with trehalose pretreatment (Sun et al. 2011). The elevation of melting temperature, combined with no change in glass transition temperature of the dried lens tissue with trehalose pretreatment in this study, indicates that the tissue would stay in the glassy state for a wider range of temperature under the presence of trehalose. These facts might explain the protective effect of trehalose on the tissues or cells in desiccation. A rise in melting temperature by trehalose for lyophilized proteins in a preceding study also supports this line of reasoning (Attanasio et al. 2007).

One possible limitation in this study would be that the residual water content in each dried tissue might show trivial difference among the samples even under the circumstances that the lens tissues were dried thoroughly in a desiccator until no weight change. The larger standard deviation for glass transition temperature of saline or cyclic tetrasaccharide-pretreated samples was noted in contrast with the smaller standard deviation for glass transition temperature of trehalose or maltose-pretreated samples. The large variation of the glass transition temperature, measured in the condition of saline or cyclic tetrasaccharide pretreatment, might be attributed to the difference in residual water content among the dried tissue samples. Preceding studies on thermal analysis of isolated proteins also mentioned that trehalose would keep moisture in lyophilized proteins and might influence the measurement of glass transition temperature (Mazzobre and Buera 1999; Hedoux et al. 2009).

## Conclusions

The glass transition temperature of isolated porcine lens as a biological tissue in the dried condition could be measured by differential scanning calorimetry. We could not detect any significant change in the glass transition temperature of the dried lens tissues with pretreatment by different sugars in the different concentrations. However, trehalose pretreatment appeared to make the glass transition temperature more noticeable on the thermogram, compared with other sugar treatment. The presence of trehalose in the drying condition might lead to more regular alignment of biological macromolecules, and thus, would result in more apparent detection of the glass transition temperature. Thermal analysis of the whole tissue, such as the lens in this study, by differential scanning calorimetry, would lead to the elucidation of mechanism for cataract formation (Biro et al. 2006) and also to the development of methods to preserve cells and tissues in the drying or frozen condition (Wolkers et al. 2001, 2002; Li et al. 2007; Orlien et al. 2003; Lynch et al. 2011).

## Methods

Enucleated porcine eyes were obtained from a local slaughterhouse, stored at 4°C, and used within 6 hours. A 10-mm-wide incision at the midperiphery of the eyeball was made to extract the lens. The vitreous gel and the lens were squeezed out of the eyeball by being pushed with fingers gently. The released lenses were placed in saline (0.9% sodium chloride) without direct touching and washed for 10 min. The lenses were transferred with a spoon to wells of a 24-well multidish containing either saline, trehalose (Hayashibara, Inc., Okayama City, Japan), maltose, or cyclic tetrasaccharide (Hayashibara) at 100 mM or 1000 mM concentration dissolved in saline (Matsuo 2005; Sun et al. 2011). The lenses were incubated for 150 minutes at room temperature, and then, all solutions were removed out of the wells by aspiration. The lenses were dried at room temperature in a desiccator until the weight showed no change. It took about eight days to reach this state of dryness (Sun et al. 2011).

Before proceeding to differential scanning calorimetric measurements, the dried lenses were ground into fine powder on a workbench with a metal bar in order to make samples have close contact with the bottom of an aluminum platform pan. Each sample with the weight around 20 mg was transferred onto an aluminum platform pan by a medicine spoon, and then the pan was sealed by a sample press kit. We used an empty aluminum pan as a reference for differential scanning calorimetric measurements. The measurements were carried out on a Seiko DSC-220, assembled with an SSC-5300 thermal controller (Seiko Instruments Inc., Chiba, Japan). The samples were cooled at a scan rate of 10 K/min to −80°C, held for two minutes at −80°C, and then heated at a scan rate of 10 K/min from −80°C to 180°C, under the atmosphere of nitrogen at a flow rate of 50 mL/min to stabilize the temperature in measurements (Sun et al. 2011).

The glass transition temperature was identified as a changing point of the slope of curvature on the thermogram. For statistical analysis, the significance was analyzed with Kruskal-Wallis test and a significant level was set at less than 0.05.
